# The Comprehension of Grammatical Structures in a Pediatric Population with ASD and Epilepsy: A Comparative Study

**DOI:** 10.1007/s10803-024-06291-9

**Published:** 2024-02-23

**Authors:** Alejandro Cano Villagrasa, Nadia Porcar Gozalbo, Beatriz Valles González, Miguel López-Zamora

**Affiliations:** 1 Facultad de Ciencias de la Salud, Universidad Internacional de Valencia, Valencia, Spain; 2 Departamento de Psicología Evolutiva y de la Educación, Facultad de Psicología y Logopedia, Universidad de Málaga, Málaga, Spain

**Keywords:** ASD, Epilepsy, Childhood, Auditory comprehension, Morphosyntax

## Abstract

Autism Spectrum Disorder (ASD) and epilepsy represent a comorbidity that negatively influences the proper development of linguistic competencies, particularly in receptive language, in the pediatric population. This group displays impairments in the auditory comprehension of both simple and complex grammatical structures, significantly limiting their performance in language-related activities, hampering their integration into social contexts, and affecting their quality of life. The main objective of this study was to assess auditory comprehension of grammatical structures in individuals with ASD and epilepsy and compare the results among the three groups. A non-experimental cross-sectional study was designed, including a total of 170 participants aged between 7 and 9 years, divided into three groups: a group with ASD, a group with epilepsy, and a comorbid group with both ASD and epilepsy (ASDEP). The comprehension of grammatical structures was assessed using the CEG and CELF-5 instruments. Statistical analyses included MANOVA and ANOVA to compare scores between groups to verify associations between study variables. The results indicate that the group with ASD and epilepsy performed worse compared to the ASD and epilepsy-only groups, respectively. Additionally, a significant and directly proportional association was observed among all variables within the measures of grammatical structure comprehension. The neurological damage caused by epilepsy in the pediatric population with ASD leads to difficulties in understanding oral language. This level of functioning significantly limits the linguistic performance of these children, negatively impacting their quality of life and the development of core language skills.

## Introduction

The pediatric population with Autism Spectrum Disorder (ASD) and comorbid neurological conditions such as epilepsy exhibits a highly heterogeneous linguistic profile (Wiklund & Laakso, 2021). Deficits in language skills represent one of the most relevant factors determining the clinical severity of children with ASD (Hirota & King, 2023). However, when an individual with ASD also presents with epilepsy, competencies in expressive and receptive language manifest greater difficulty compared to when only one of these two disorders is present (Braconnier & Siper, 2021). Linguistic alterations in children with ASD and epilepsy can be described as a highly variable continuum, where on one hand, there are minimally verbal children (Tuchman et al., 2010) or those who do not acquire verbal language (in variable percentages of up to 50% according to different studies), and on the other hand, there are children with a level of linguistic form development that can be considered appropriate (Duffy et al., 2013), but pragmatically inadequate (Roberts et al., 2008). Within this spectrum, highly variable profiles of linguistic development can be found (Mukherjee, 2017), with children exhibiting mild language delay (Davidson & Weismer, 2017), children with severe language disorders (Eigsti et al., 2015), and those who, despite reaching their early development milestones, such as first word production or usage between 12 and 18 months, subsequently experience stagnation or regression in their language development (Ricketts, 2011).

Although communication and language in children with ASD have been extensively researched (Davidson et al., 2018; Naigles, 2013; Torrens & Ruiz, 2021), when this disorder is accompanied by epilepsy, the nature and characteristics of language deficits become an evident issue that requires further investigation (Cano-Villagrasa et al., 2023). For instance, according to the DSM-5 (APA, 2013), ASD exhibits significant alterations in the pragmatic-social dimension of expressive language. Its diagnostic criteria emphasize difficulties in establishing functional conversations and interacting with the environment, particularly in contexts where linguistic demands on the individual with ASD are higher. However, the DSM-5 diagnostic criteria do not provide a detailed description of the linguistic alterations that a child with a diagnosis of both ASD and epilepsy might present. Some authors consider ASD and epilepsy as potentially comorbid conditions (Ali, 2018; Ricketts, 2011; Tran et al., 2020) and suggest that these clinical profiles are associated with more pronounced disruptions in expressive language development (Eigsti et al., 2015). These disruptions include difficulties in phonological, semantic, morphosyntactic, and pragmatic functioning, in addition to receptive language impairments involving comprehension and following complex instructions with multiple elements (Saban-Bezalel & Mashal, 2019).

Delving into linguistic competencies related to language comprehension, most published studies report that in ASD and epilepsy, receptive language is more affected than expressive language (Asberg, 2010; Henry & Solari, 2020). However, this finding has not been fully confirmed (Roemer et al., 2019). Furthermore, the question of the discrepancy between receptive and expressive competencies is still unclear in terms of whether it should be considered a potential marker of ASD (Saban-Bezalel & Mashal, 2015) or if both receptive and expressive language difficulties are comorbid with ASD and epilepsy (Cano-Villagrasa et al., 2023).

Difficulties in language comprehension in children with ASD and epilepsy are part of a broad and complex spectrum characterized by pragmatic deficits (Eberhardt & Nadig, 2018) and executive functioning impairments that can affect verbal comprehension (Asberg et al., 2010). According to Tager-Flusberg et al. (2009), language comprehension problems in the presence of this comorbidity are particularly evident in everyday situations rather than during single-word comprehension tasks. Children with ASD and epilepsy have impairments in their ability to decode relevant contextual cues and deficits in social attention (Svindt & Surányi, 2021), while typically developing children can identify or select salient sensory and social stimuli relevant for both comprehension and communication from an early age (Boucher, 2012).

Grammatical comprehension, as a specific receptive language skill required to decode verbal messages in interactions (Naigles & Tovar, 2012), represents a crucial research target in the field of ASD and epilepsy (Kalandadze et al., 2022). However, it is worth noting that few studies assess the comprehension of individuals with ASD and epilepsy through specific tasks that evaluate differences in their grammatical comprehension skills.

Several research findings suggest that syntactic deficits are apparent across individuals on the spectrum, including among older adolescents with ASD and epilepsy who have average or above-average cognitive abilities (Eigsti & Bennetto, 2007; Gonzalez-Barrero & Nadig, 2019; Loukusa et al., 2014). However, syntactic deficits tend to be localized rather than global and vary among individuals with ASD and epilepsy. For instance, some children with these two disorders display deficiencies in the comprehension of past tense verbs (Kelley et al., 2006), while others exhibit alterations in morpheme comprehension (Tovar et al., 2015). Similar to the semantic component, research suggests variability in syntactic language skills exhibited by individuals with ASD and epilepsy (Eigsti & Bennetto, 2007).

Some authors hypothesize that this variability can be explained by cognitive abilities and the severity of symptoms in ASD and epilepsy, as individuals with these conditions show deficits in syntactic language (Roberts et al., 2010; Shulman & Guberman, 2007). However, other authors have found that even among individuals with ASD and epilepsy who have better cognitive abilities, most still demonstrate specific syntactic deficits, such as the use of simplified phrases, limitations in the comprehension of complex grammatical structures, and challenges with verb tense conjugation and inflection (Eigsti & Bennetto, 2007; Kelley et al., 2006).

The discrepancy in findings likely reflects the interplay between ASD and epilepsy, as several studies have reported that this population experiences difficulties in comprehending grammatical structures related to passive voice or those with a high degree of inferential content (Roberts et al., 2010; Shulman & Guberman, 2007). Given the limited literature examining the comprehension of morphosyntactic structures in the pediatric population with ASD and epilepsy, along with methodological limitations in research in this area, there is a need to expand research in this domain to gain a better understanding of syntactic language in these disorders and the influence of syntactic language skills on other aspects of psychosocial functioning for children with ASD and epilepsy.

Therefore, the assessment of receptive skills in children with ASD and epilepsy is of crucial clinical importance for defining their language profile more clearly (Ge et al., 2023). It is considered a fundamental prognostic marker for the development of linguistic competencies (Garrido et al., 2015). In fact, children with ASD and epilepsy who have minimal ability to emit verbal information and communication exhibit greater severity of autistic symptoms and overall worse clinical outcomes (Baixauli et al., 2020) than those with only one of the two disorders, for whom the outcome might be more satisfactory (Venker et al., 2016).

For these reasons, the main objective of this study was to evaluate competencies in the comprehension of grammatical structures in the Spanish language in a group of minors diagnosed with ASD and epilepsy. Additionally, a series of specific objectives were established, including: (I) comparing performance in grammatical structure comprehension tasks, and (II) examining the relationship between the sub-scales of grammatical structure comprehension among the three participant groups. Based on the empirical evidence in the scientific literature, it was hypothesized in this study that participants diagnosed with both ASD and epilepsy would perform worse in comprehension tasks compared to the group diagnosed with ASD, followed by the group composed of individuals diagnosed with epilepsy.

## Method

### Participants

All participants diagnosed with ASD or epilepsy underwent evaluation in the Mental Health Unit and Pediatric Neurology service attached to their medical reference center by a multidisciplinary professional team. In these centers, a diagnostic screening assessment was conducted using the M-CHAT-R questionnaire (Robins et al., 2004). Children who scored in a way that raised suspicion underwent the ADOS-2 (Lord et al., 2008) and ADI-R (Rutter et al., 2006) protocols to confirm the diagnostic suspicion of ASD. Additionally, participants received a neurological evaluation from the neuropediatric team, which included a neurological assessment using Magnetic Resonance Imaging, sleep-deprived Electroencephalogram, and genetic tests to help confirm the diagnosis.

Finally, the following inclusion and exclusion criteria were established. Inclusion criteria were: (I) being between 7 and 9 years old, (II) having a diagnosis of ASD issued by a public hospital or health center, and (III) undergoing rehabilitation treatment at the rehabilitation clinic. Exclusion criteria were: (I) having any motor or sensory disease or disorder that hinders or prevents proper study performance, (II) not exhibiting any communication or language by 5 years of age, and (III) having mild, moderate, severe, or profound intellectual disability or an IQ score below 75 as measured by the WISC-V test (Wechsler, 2015).

### Instruments and Materials

#### “CEG. Test de Compresión de Estructuras Gramaticales”

The *CEG. Test de Comprensión de Estructuras Gramaticales* (CEG; Mendoza et al., 2005) is an assessment instrument that measures the comprehension of statements and sentences with varying levels of morphosyntactic complexity in the Spanish language. It is standardized for the Spanish population between the ages of 4 and 12. This test consists of 80 multiple-choice items (four answer alternatives) distributed across 20 blocks of four items each, representing the most representative grammatical structures in the Spanish language. The internal consistency of the test has been studied using Cronbach’s alpha coefficient, and a reliability index of 0.9096 has been obtained. The reliability obtained for each age group was as follows: 4 years: 0.825; 5 years: 0.779; 6 years: 0.866; 7 years: 0.797; 8 years: 0.828; 9 years: 0.807; 10 years: 0.794; and 11 years: 0.842.

#### CELF-5. Clinical Evaluation of Language Fundamentals − 5

The Clinical Evaluation of Language Fundamentals − 5 (CELF-5; Wiig et al., 2013) is an individual clinical assessment instrument designed to identify, diagnose, and monitor language and communication disorders in children and adolescents aged 5 to 15 years. This individual application instrument aims to evaluate the user’s strengths and weaknesses in different aspects and dimensions of language in Spanish. This instrument consists of 14 subscales, but only two were used in this research: Sentence comprehension and Execution of instructions. These two scales are scored with 0–1 points, with 0 indicating the absence of the skill and 1 indicating its presence, except for the linguistic profile questionnaire, which consists of a structured response on a Likert-type scale of 0–4, where a score of 0 is associated with “never” and 4 with “always.” The internal consistency of the CELF-5 obtained by Cronbach’s alpha coefficient is 0.901. According to the study by Denman et al. (2017), the reliability of the test is between 72.4 and 66.7, which makes it an instrument with good psychometric quality evidence and its use is recommended.

### Procedure

For the completion of this study, first, approval was obtained from the Ethics Committee of the Universidad Católica San Antonio de Murcia (UCAM), with the code: CE052206. Next, the participant groups were configured, following the inclusion and exclusion criteria previously established for this study, and they were grouped into three experimental groups. In this way, an evaluation of the participants included two 60-minute sessions. The data from the measurement instruments were stored in protected databases, which were later analyzed by the research group members, checking the fulfillment of the research hypotheses.

### Design and Data Analysis

The statistical analysis of this non-experimental cross-sectional study was conducted using the Statistical Package for the Social Sciences (SPSS) software, version 23.0 for Windows, developed by IBM, for statistical analysis in this study. Two different statistical designs were used to carry out the analyses. First, the Kolmogorov-Smirnoff test was used to examine the normality of the distribution of the variables that make up the study, which was found to meet the normality assumption. Next, multivariate analysis of variance (MANOVA) was used to observe the relationship between the variables related to the comprehension of different types of grammatical structures in Spanish. This allowed for exploring the differences among the groups that make up the sample. Subsequently, analysis of variance (ANOVA) tests was performed to observe individual differences in each of the variables in the three groups. Finally, to control for Type I error, the Holm-Bonferroni correction (Holm, 1979) was applied to the statistical analyses conducted. Subsequently, post hoc analyses for multiple comparisons between the three groups (ASD, Epilepsy, and ASD and epilepsy) were performed using Bonferroni’s method (Bonferroni, 1936).

## Results

A total of 170 participants aged between 7 and 9 years were selected and divided into three groups: the ASD group (*n* = 57), the ASD and epilepsy group (*n* = 53), and the epilepsy-only group (*n* = 60). The ASD group (ASD) consisted of 36 boys and 21 girls (M_age_ = 8.1; SD = 0.9) diagnosed with grade 1 ASD. The Epilepsy group (EP) comprised 41 boys and 19 girls (M_age_ = 8.6; SD = 0.4). The ASD and epilepsy group (ASDEP) consisted of 27 boys and 26 girls (M_age_ = 7.8; SD = 0.8) who also had a diagnosis of grade 1 ASD and were evaluated and diagnosed at their medical reference center. All participants were native Spanish speakers. Table 1 summarizes the main characteristics of the participants who comprised the study’s sample group:

**Table 1:** Characteristics of the study participants

Characterization of the participants			
		N	Percentage
Sex	Male	104	61.2
	Woman	66	38.2
Age	7 years old	47	27.6
	8 years old	61	35.9
	9 years old	62	36.5
Diagnosis	ASD	57	33.5
	ASD + Epilepsy	53	31.2
	Epilepsy	60	35.3
Years of treatment	2 years	34	20.0
	3 years	73	42.9
	4 years	63	37.1
Grade of incapacity	Less than 33%	52	29.1
	Between 33% and 66%	74	40
	More than 66%	44	30.9
Educational supports at school	Hearing and Language Teacher	41	30.6
	Teacher of Hearing and Language and Therapeutic Pedagogy	87	51.2
	Hearing and Language Teacher, Therapeutic Pedagogy and Educator	42	24.7
Gestation weeks	30–35	52	30.6
	35–40	87	51.2
	More than 40	31	18.2
Apgar	With risk	77	45.3
	Intermediate	63	37.1
	Normal	30	17.6
Intellectual disability	No presence	123	72.4
	Mind	47	27.6

With respect to the results of the analyses, first, the Kolmogorov-Smirnoff test was performed, and its significance level was found to be greater than 0.05, indicating that there is insufficient empirical evidence to reject the null hypothesis, which suggests that the distribution of the participants’ scores follows a normal distribution. In addition, a descriptive analysis was performed to determine the mean scores (M) and standard deviation (SD) for each of the variables included in the study. Finally, the MANOVA conducted to assess differences in measures of grammatical structure comprehension among the ASD, Epilepsy, and ASD with Epilepsy groups revealed the presence of statistically significant differences (Wilks’ Lambda = 0.013, F(2,167) = 81.031, *p* < 0.001, η^2P = 0.888). As can be seen in Table 2, the variables in which significant differences were obtained included Inferences, Active Structure, Passive Structure, Declaratives, Reflexives, Datives, Interrogative, Exclamatory, Dubitative, Desiderative, Exhortative, Personal, Impersonal, Phrase Comprehension, and Instruction Execution (*p* < 0.05). The results of the ANOVAs related to the performance in the comprehension of grammatical structures are presented in Table 2.

**Table 2:** Differences among the measures of the groups: Autism Spectrum Disorder (ASD), Epilepsy (EP), and ASD with epilepsy (ASDEP) in the comprehension of grammatical structures from CEG and CELF-5 tests

Comprehension grammatical structures		ASD (n = 57)			EP (n = 60)			ASDEP (n = 53)			F _(2,167)_	η^2^_P_	Differences between groups
		M	SD		M	SD		M	SD				
**Variables included in the CEG test**													
Inferences		8.53	2.458		13.95	1.926		3.85	1.598		349.697^*^	0.807	ASDEP*<ASD*<EP*
Active structure		8.39	2.313		13.73	2.049		3.23	1.728		370.045^*^	0.816	ASDEP*<ASD*<EP*
Passive structure		8.32	2.293		14.10	2.121		3.87	1.744		347.017^*^	0.806	ASDEP*<ASD*<EP*
Declaratives		8.33	2.030		14.02	2.029		3.32	1.837		416.255^*^	0.833	ASDEP*<ASD*<EP*
Reflective		8.42	2.236		13.85	2.057		3.45	1.749		370.252^*^	0.816	ASDEP*<ASD*<EP*
Datives		8.46	2.164		14.00	1.957		3.23	1.739		423.763^*^	0.835	ASDEP*<ASD*<EP*
Interrogative		8.23	2.053		14.27	1.831		3.38	1.643		490.018^*^	0.854	ASDEP*<ASD*<EP*
Exclamative		8.46	2.465		14.00	2.099		3.45	1.716		348.517^*^	0.807	ASDEP*<ASD*<EP*
Doubtful		8.98	2.272		13.73	1.840		3.68	1.718		369.841^*^	0.816	ASDEP*<ASD*<EP*
Desiderative		8.19	2.560		14.00	2.131		3.74	1.631		324.219^*^	0.795	ASDEP*<ASD*<EP*
Exhortative		8.77	2.260		13.75	2.055		3.70	1.917		327.132^*^	0.797	ASDEP*<ASD*<EP*
Personal		8.72	2.234		14.23	1.826		3.72	1.634		424.304^*^	0.836	ASDEP*<ASD*<EP*
Impersonal		8.60	2.404		14.00	1.957		4.00	1.687		339.093^*^	0.802	ASDEP*<ASD*<EP*
**Variables included in the CELF-5 test**													
Sentence comprehension		8.88	2.398		14.12	2.210		3.81	1.688		329.722^*^	0.798	ASDEP*<ASD*<EP*
Execution of instructions		9.11	2.425		14.35	1.921		3.77	1.552		391.354^*^	0.824	ASDEP*<ASD*<EP*

*p < 0.05

As can be observed in Table 2, Bonferroni post-hoc tests showed statistically significant differences between the ASD, Epilepsy, and ASD with Epilepsy groups in all variables included in the measures of grammatical structure comprehension (*p* < 0.001). Thus, the scores obtained by the Epilepsy group were higher than those of the ASD group, and these were higher compared to the ASD with Epilepsy group.

**Fig. 1:**
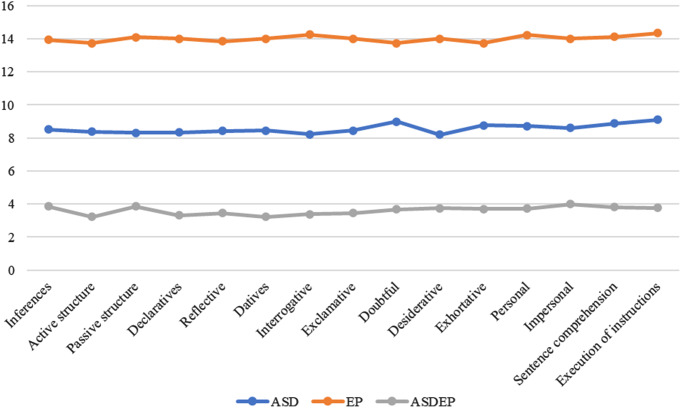
Comparison of grammatical structures of comprehension between ASD, epilepsy and ASD with epilepsy groups from CEG and CELF-5 tests

To provide a visual perspective of the mean scores obtained by the three groups in the study, a graph (Fig. 1) was created, which displays a descriptive comparison of the means obtained in each of the variables related to the comprehension of grammatical structures.

As can be observed in Table 2, the variables included in the measurements of grammatical structure comprehension show a statistically significant association and a directly proportional trend (*p* < 0.001). These results have been established with a 99% confidence interval (*p* = 0.001).

## Discussion

As previously described, the main objective of this study was to explore the differences in the performance of grammatical structure comprehension among three groups of participants diagnosed with ASD, epilepsy, and ASD with epilepsy. The research hypothesis posited that the group of participants diagnosed with epilepsy alone would perform better than the group of participants with ASD, followed by those with the comorbidity of ASD and epilepsy.

The results obtained in our study indicate that participants with epilepsy demonstrate better performance than the other two groups of participants, confirming what was initially proposed in the research hypothesis for this study. Significant differences in grammatical structure comprehension skills exist between the groups. These data cannot be confirmed by other research because there are no studies that compare performance in grammatical structure comprehension in these three disorders. However, these results can be corroborated with other studies conducted with these groups independently (Asberg, 2010; Baixauli et al., 2020; Eigsti et al., 2007; Eberhardt & Nadig, 2018; Henry & Solari, 2020; Saban-Bezalel & Mashal, 2015), which confirm their contribution to the communicative problems presented by individuals with these three disorders, with varying performance in these skills depending on the specific type of diagnosis.

Regarding the pediatric population with epilepsy, there is no consensus on the specific grammar-related skills that may be affected. Some tasks that have been reported as impaired in some studies (Durrleman et al., 2015; Su et al., 2018) appear preserved in others (Bangert et al., 2019; Maltman et al., 2023). However, there seems to be a higher prevalence of poorer performance in tasks involving syntactic skills, such as completing and producing sentences, understanding ambiguous sentences, sentence-picture matching, and comprehending complex syntactic structures (Bulteau et al., 2015; Skotko et al., 2008). Although previous studies have extensively analyzed comprehension skills related to morphosyntax in these children, there is no significant data that allows for the establishment of a specific linguistic profile (Cohen & Le Normand, 1998; Ménard et al., 2000). In terms of verbal comprehension, they exhibit difficulties in understanding verbal messages, often requiring message repetition, providing correct answers/information, and reformulating messages for optimal retention and comprehension (Teixeira & Santos, 2018). In our study, we observed poor performance in variables related to grammatical structure comprehension, especially those involving or related to executive functioning skills, such as understanding inferences, reflexive statements, and executing instructions. One possible explanation for these difficulties may be attributed to the damage present in this population in areas such as the frontal lobe and the networks connecting this lobe to the temporal lobe, which is the primary area responsible for oral language comprehension (Ballester-Plané et al., 2018). People with epilepsy have slowed processing speed, which also hampers effective comprehension of grammatical statements (Caplan et al., 2009). However, even though this population experiences difficulties in these skills, their performance will be superior to what is observed in other neurodevelopmental disorders such as ASD (Debiais et al., 2007).

In the case of participants diagnosed with ASD, current scientific literature indicates that these clinical profiles are associated with impairments in both language expression and comprehension. For example, Eigsti & Bennetto (2007) observed grammatical disorders in the majority of children with ASD, aged 9–17 years, in grammatical judgment tasks. In our study, the structures that are most challenging for children with ASD are those that do not follow the common structural order, subject-verb-predicate (SVP), such as coordinated sentences or relative clauses, in which all these children failed. Conversely, the easiest structures are those of SVP, with a 60% success rate, followed by attributive structures, with a 30% success rate (Barsotti et al., 2020). Swensen et al. (2007) also state that children with ASD understand sentences in this SVP order before producing continuous speech. These results demonstrate the difficulties that children with ASD face, limiting their understanding of verbal messages from the main agents in their environment, hindering functional communication, and engaging in more complex conversations beyond their personal interests or motivations. Therefore, difficulties in verbal comprehension have a negative impact on the communicative skills and quality of life of individuals with ASD, severely affecting their inclusion in developmental contexts (Tovar et al., 2015).

Finally, the pediatric population with both ASD and epilepsy displays severe impairments in receptive language skills, including the comprehension of grammatical structures in oral language (Cano-Villagrasa et al., 2023). To date, no research on grammatical structures in Spanish-speaking children with both ASD and epilepsy is available. Therefore, the results indicate that language comprehension, especially grammatical structures, is one of the areas of weakness in both ASD and epilepsy that may contribute to explaining the observed communication deficits. However, there is very little evidence regarding grammatical impairments, and this remains an open area for research (Kanner & Bicchi, 2022). In the present study, the group of participants with both ASD and epilepsy displayed the lowest performance in grammatical structure comprehension tasks among the three groups in this investigation. One of the most plausible explanations for these results is that the presence of epilepsy, in addition to the diagnosis of ASD, leads to increased difficulties in language skills, especially those related to receptive language (Stefanski et al., 2021). With impaired executive functioning, as well as in linguistic dimensions such as lexicon, morphology, and pragmatics, it is logical to assume that verbal comprehension of grammatical structures is also compromised, primarily due to difficulties in following conversations and instructions. This negatively impacts their language skills, resulting in poorer performance in such tasks compared to other profiles, such as those in the epilepsy group and the ASD group (Montouris et al., 2020). Therefore, although studies like Saban-Bezalel and Mashal (2015) suggest that the relationship between auditory comprehension and the severity of linguistic symptoms in ASD is not clear, the present study confirms that linguistic comprehension is a predictor of language difficulties in ASD.

The study has certain limitations that should be taken into account. Firstly, the small sample size raises questions about the generalizability of the results to a larger population. Additionally, variability in the ages of the participant groups could introduce elements of uncertainty in the results, as language abilities tend to evolve with age. Heterogeneity in individual conditions within each group could also influence the observed results. It is noteworthy that external factors, such as linguistic environment and prior education, were not taken into account, which could have had an impact on the participants’ performance. Finally, the specific methodology used in the tests may limit comparability with other similar studies. Despite the mentioned limitations, the study has several strengths worth considering. The formulation of a clear and precise hypothesis provided a specific direction for the research. The results obtained represent a significant contribution to the scientific corpus, shedding light on differences in grammatical comprehension in neurological contexts. Detailed analyses of the particular difficulties of each group enrich our understanding of the linguistic challenges they face. The multidisciplinary perspective of the study, considering both neurological conditions and language skills, offers valuable implications for clinical and educational practice, suggesting ways to address these difficulties from multiple areas.

In conclusion, this study highlights the diversity in grammatical structure comprehension among different participant groups: those with epilepsy, ASD, and ASD with epilepsy. The results support the notion of better performance in the epilepsy group, followed by the ASD group, while the group with both conditions exhibited the lowest performance. These discrepancies have significant implications for communication and the quality of life of those affected. Furthermore, they emphasize the need for future research to delve into the comprehension of grammatical impairments in these disorders and their relationship with other cognitive and linguistic aspects. Ultimately, these findings support the importance of developing personalized therapeutic approaches and educational programs to address the specific needs of these groups of children.
